# Perceptions of Adult Obesity Education: A Pilot Study

**DOI:** 10.1177/23821205241269371

**Published:** 2024-10-01

**Authors:** Seleda Ann Williams, Cara Marie Sandholdt, Jeffrey Robert Fine, Kougang Anne Mbe

**Affiliations:** 1 Departments of Internal Medicine & Public Health Sciences, 8789 University of California Davis Health, Sacramento, CA, USA; 2 Betty Irene Moore School of Nursing, 8789 University of California Davis Health, Sacramento, CA, USA; 3 Department of Biostatistics, Epidemiology and Research Design, 8789 University of California Davis Health, Sacramento, CA, USA

**Keywords:** Obesity, medical education, training, primary care, weight management, nutrition, bariatric surgery, behavioral health

## Abstract

**Objectives:**

This pilot research study, conducted at a large academic healthcare facility, used mixed methodology to (1) administer a survey to a group of primary care trainees and faculty and (2) conduct key informant interviews with the program directors, or their delegates of these primary care training programs, so as to gain insight into respondents’ perceptions about their training on adult obesity. To maintain confidentiality of the key informants, they were defined as “Administrators.” Faculty and trainees were from family medicine and internal medicine residency programs, as well as family nurse practitioner and physician assistant training programs.

**Methods:**

This study used a quantitative survey and four qualitative key informant (Administrator) interviews. Descriptive statistics, χ^2^, or Fisher exact tests were used to analyze select survey responses. Administrator interviews were analyzed with thematic analysis.

**Results:**

Survey respondents (n = 75) included primary care trainees (n=34), faculty (n=30), other (n=2), did not answer (n=9). Surveys indicated that additional training is needed for bariatric surgery, weight loss medications, and clinical nutrition. The three highest ranked topics in the surveys on adult obesity were basic nutrition, behavioral weight management, and a rotation on adult obesity. Most agreed on the need for interprofessional collaboration, a centralized obesity treatment center, and an introductory obesity course. Key themes from the four Administrator interviews revealed the need: for more training; to build upon current curriculum; use innovative technology; fiscal challenges; and time management.

**Conclusions:**

Both faculty and trainees perceive that academic and clinical training on adult obesity is inadequate, and that trainees need more education on such topics as nutrition, physical activity, behavioral health, antiobesity medications, and bariatric surgery. Competency to treat varied by topic. It also showed that more interprofessional collaboration and a centralized obesity treatment center are needed. Recommendations included integrating modular units about obesity into already established primary care training programs and providing additional resources.

## Introduction

Even though almost three quarters of adult Americans are either overweight or obese (73.8%),^1^ there still remain significant gaps in training healthcare professionals about how to assess and treat adult obesity.^1–6^ Moreover, adult obesity continues to rise in the United States and clearly has significant race/ethnic disparities.^1,7,8^ Given the correlation of increased morbidity and mortality with increasing body mass index (BMI), it is important to understand how we educate our healthcare providers to care for people who are obese while being cognizant of any implicit weight bias.^9–12^ There are limited studies looking at how primary care providers are trained to assess and treat people who are obese,^13–15^ and few studies have examined how to incorporate curriculum on obesity into already crowded training programs.^5,6,14,16^ In addition, recent studies indicate that team-based obesity care may improve treatment outcomes and that more research is needed in this area.^16–19^

This novel exploratory pilot study attempts to delve deeper into where the gaps exist at a teaching university and identify areas for future research. It is a mixed methodology pilot research study which examined how adult obesity training and treatment services could be improved in a large academic healthcare facility. We postulated that there are opportunities for improvement in primary care medical education and curriculum development on the assessment and treatment of adult obesity. We investigated how a large public university and affiliated health center, teaches primary care trainees about adult obesity. For purposes of our research, “primary care trainees” included respondents from family medicine, internal medicine, nurse practitioner, and physician assistant programs.

Our research group used a survey and key informant interviews to conduct this quantitative and qualitative investigation. A set of unique key informant interview questions were created to explore the institution's concerns and needs related to obesity education and treatment services. To maintain confidentiality, the invited key informant interviewees, who were either program directors or their delegates, were defined as “Administrators.”

The researchers also utilized the Qualtrics^XM^ survey (survey) tool to implement a set of original survey questions tailored to the specific hypotheses of the research study. The survey created questions that addressed interprofessional, cross disciplinary obesity-related questions including nutrition, physical activity, epidemiology, antiobesity medications, bariatric surgery, weight management, and treatment services for people who are obese.

We hypothesized that: (1) primary care faculty, residents and students would indicate the need for additional training in the clinical assessment and treatment of adult people with obesity. (2) adult obesity treatment services could be improved. (3) faculty and trainees would support interprofessional collaboration for treatment of adult obesity. We developed a set of nine “SMART” (specific, measurable, achievable, relevant, time-based) objectives/aims to prove these hypotheses.

Our university and affiliated health center have a strong legacy in primary care training and interprofessional education; however, it has been difficult to translate this research expertise into providing adequate training on adult obesity to its primary care trainees. This study attempts to find out why.

## Methods

To our knowledge, this pilot research study is the first of its kind at our institution. It was conducted at the University of California Davis Health as part of a faculty development interprofessional scholars’ program. It used both quantitative and qualitative data to evaluate its hypotheses and aims. Through the development of a logic model, and literature reviews the hypotheses were developed. The research team collaborated with chronic disease experts in the institution's departments of Internal Medicine; Family Medicine; Endocrinology, Diabetes and Metabolism; and the Department of Women's Cardiovascular Medicine. These steps guided the development of a voluntary “Qualitrics^XM^” web-based survey and key informant interviews (please see Supplement 1 for a copy of all the survey questions and Supplement 2 for all the key informant questions) (please see Supplement 3 for Key Informant Interviews theme tables). These were designed to identify gaps in training on adult obesity and to assess respondents’ self-perceptions about the adequacy of their training on obesity, levels of confidence, interprofessional collaboration and other parameters as described in detail in the survey and interview design sections below. Written informed consent was obtained for both the survey and key informant interviews.

The reporting of this study conforms to the equator network search guidelines for two search guidelines, one for our key informant interviews and one for our survey. The COREQ 32-Item Checklist^20^ was completed for our key informant interviews (please see Supplement 4); and the CRISP checklist^21^ for our primary care survey (please see Supplement 5).

The study population for the 2020/2021 academic year, consisted of “primary care” Internal Medicine (IM)/Family and Community Medicine (FM) faculty and third-year residents; as well as Family Nurse Practitioner (FNP)/Physician Assistant (PA) faculty and second year Master's students from the School of Nursing (SON).

The survey was distributed by the researchers via e-mail distribution lists to 191 primary care faculty, residents, and students, with lists provided by the respective program Directors or delegates (Administrators). E-mails included instructions and a hyperlink to the consent form and confidential survey.

Research protocols, study design, survey, and key informant interview questions were approved by our facility's Institutional Review Board (IRB).

Key informants were invited to participate from the departments of IM and FM residency programs, as well as from SON FNP and PA programs to be interviewed as Administrators. These individuals were contacted by e-mail and invited to participate with a 60-min video interview. All names were removed, and participants were referred to as “Administrators.”

Literature reviews using PubMed were conducted using the academic institution's library system prior, during, and after the intervention.

### Inclusion Criteria

Researchers distributed the survey by e-mail distribution lists our institution's IM/FM residents and faculty, as well as SON FNP/PA students and faculty. Four Key Informant Administrators from our institution were interviewed from the IM/FM residency programs and the SON FNP/PA programs.

### Exclusion Criteria

Any faculty, resident, or FNP/PA student not in the defined in our institution's primary groups were excluded from the surveys or key informant interviews. A drop-down box question on the survey excluded any respondents that did not meet the study inclusion criteria. Residents and faculty that were not with the IM/FM residency or SON FNP/PA program were not invited to take the survey. Other primary care groups were not included due to time and resources.

### Survey Design

#### Qualtrics^XM^ survey

The researchers utilized the Qualtrics^XM^ survey tool to develop a set of original survey questions. The survey was anonymous and confidential and was approved by the institution's IRB committee. The survey was titled: “Adult Obesity Primary Care Survey” (survey) and utilized a variety of question formats, including demographics, yes/no answers, Likert matrix tables, and text box questions (please see Supplement 1 for all the survey questions). After participants consented to the survey there were a total of 23 questions, however, not all participants were asked all questions. Select questions, utilized forced and skip logic to ask certain questions only to faculty, or only to residents or only to FNP/PA students. The Likert matrix questions were developed to break down specific interprofessional questions related to confidence to teach or treat listing multiple cross disciplinary obesity-related topics.

Questions were asked about a variety of topics related to the assessment and treatment of adult obesity. Some included questions about the adequacy of training and confidence to treat in key areas including obesity epidemiology, weight management, nutrition, physical, behavioral health, weight loss medications, very low-calorie diets, and bariatric surgery. Survey questions reported in “Results” section include the topics: trainees’ adequacy of training; faculty confidence to teach; faculty importance of interprofessional collaboration; faculty and trainees’ confidence to treat; faculty versus trainee's confidence to treat; trainees’ attendance at clinic site rotations on obesity; faculty and trainees’ support for a centralized obesity treatment center; faculty and trainees’ need for an “Obesity 101” introductory type course; faculty ranking of needed topics for obesity training; faculty and trainees’ use of outside weight management services; and open text comments.

Please see Supplement 1 for a complete list of survey questions and survey logic.

### Survey Administration

E-mail requests were sent to the IM/FM Residency and the SON FNP/PA training Administrators requesting permission for key informant interviews and survey distribution. Dates and times were set up for administration of the survey. The last survey record was collected on May 20, 2020, IM/FM Residency and FNP/PA School Administrators sent out e-mails to faculty, residents, and students informing them that they would receive an anonymous survey. The anonymous survey was e-mailed with three reminders between April 2020 and May 2020. Respondents could only take the survey once. Surveys included consent forms and could only be completed after the respondents consented to the survey.

### Statistical Analysis

Four- or 5-point ordinal scales were used to quantify responses, such as confidence to treat and teach adult obesity, in addition to adequacy of training and treatment services. All categorical and ordinal variables were analyzed using χ^2^ or Fisher exact analyses with a 2-tailed α of .05. All statistics were conducted using SAS software for Windows version 9.4 (SAS Institute Inc).

The online Qualtrics^XM^ survey application also included summary statistical data reports which were used in this study for most of the data analysis. Our university's biostatics department also assisted with significance testing when comparing faculty versus trainee responses.

### Key Informant Interviews

Four of our institution's faculty Administrators (program directors or their delegates) from IM, FM, and SON (FNP/PA) were selected by the respective Department Chairs and invited by e-mail to participate individually in a confidential “WebEx by CISCO” webinar with the principal investigator (PI) and research assistant. Four key informant interviews were conducted and recorded by the PI and research assistant, via WebEx, from March 2020 to April 2020. All key informant interviews and recorded transcripts were de-identified with a code. All interviewees received written instructions and informed consent forms prior to the interviews, and all names were de-identified using Administrator ID numbers. The interviews were approximately one hour in length. The researchers administered a key informant interview tool to interview the Administrators. Please see Supplement 2 for a complete list of key informant interview questions. The tool included 12 questions and, if necessary, the researchers could query the interviewees further. The webinars were recorded and transcribed by the application. Question topics included: curriculum being offered on adult obesity; licensing exam requirements; recommendations on how curriculum could be improved; barriers; student and resident clinical experience with adult obesity; and other recommendations. Interviewers also took notes and compared their notes along with the transcription notes.

Key themes and recommendations were developed from the interviews and compiled into a series of tables for each interviewee (please see Supplement 3). Key informant interviewees were also invited to review summary notes to informally validate interview summaries for this qualitative portion.

## Results

### Survey Results

There were 75 of 191 respondents (39.3%). Not every respondent answered every question. A total of 75 of 77 survey respondents consented and agreed to take part in the “Adult Obesity Primary Care Survey.” Sixty-six respondents answered the demographics questions and 9 did not (Table 1).

**Table 1.** Survey Participants.

PARTICIPANTS	N
SON faculty	2
FM/IM faculty	28
FM/IM residents	23
FNP/PA students	11
Other	2
Did not answer	9
Total	75

Abbreviations: FNP, Family Nurse Practitioner; SON, School of Nursing.

#### Adequacy of training

**Figure 1.**
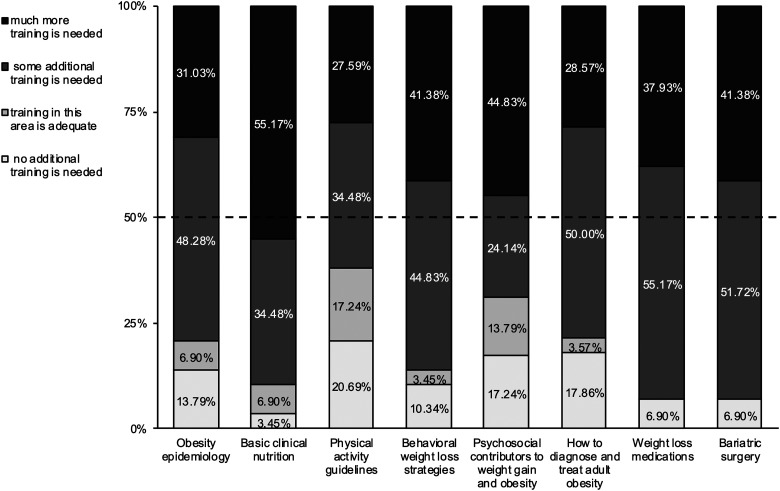
Adequacy of Training on Obesity for FNP/PA Students and FM/IM Residents.

Students and Residents (n = 29) rated the adequacy of their training in obesity for nine areas: obesity epidemiology; basic clinical nutrition; physical activity guidelines; behavioral weight loss strategies; psychosocial contributors to weight gain and obesity; how to diagnose and treat adult obesity; weight loss medications; and bariatric surgery (Figure 1). Of all these nine areas, greater than 50% of respondents indicated that “some or much more training was needed.” Of note, 0 out of 9 of these areas, related to obesity training, reached the 50% prevalence rate for “no additional training” or “training is adequate.” Furthermore, bariatric surgery had the highest percentage of respondents indicating additional training is needed at 93.1% (41.38% much more and 51.72% some), as did weight loss medications at 93.1% (37.93% much more and 55.17% some). Basic clinical nutrition had the highest percentage of respondents (89.98%) indicating additional training is needed (34.48% some while 55.17% responded much more). Physical activity guidelines had the highest rating of adequacy at 37.93% when 20.69% for “no additional training is needed” and 17.24% for “training is adequate” were combined.

Student and resident response rates for the question *“Q3.5: Please rate the adequacy of your training in adult obesity during your IM/FM residency or SON FNP/PA program” *are plotted as percentage of respondents for each of the 9 training adequacy categories assessed. A stacked plot is used to show the total percentage of responses out of 100% for “no additional training” (white), “training in this area is adequate” (light gray), “some additional training is needed” (dark gray), or “much more training is needed” (black). The dotted line indicates the 50% threshold of response rates.

Only 23.3% (7/30) of residents and students responded that they were offered any lectures or teaching modules on weight management for adult patients with obesity. Moreover, 80% (24/30) of residents and students had not attended any clinical site rotations on adult obesity and weight management.

#### Confidence to teach

**Figure 2.**
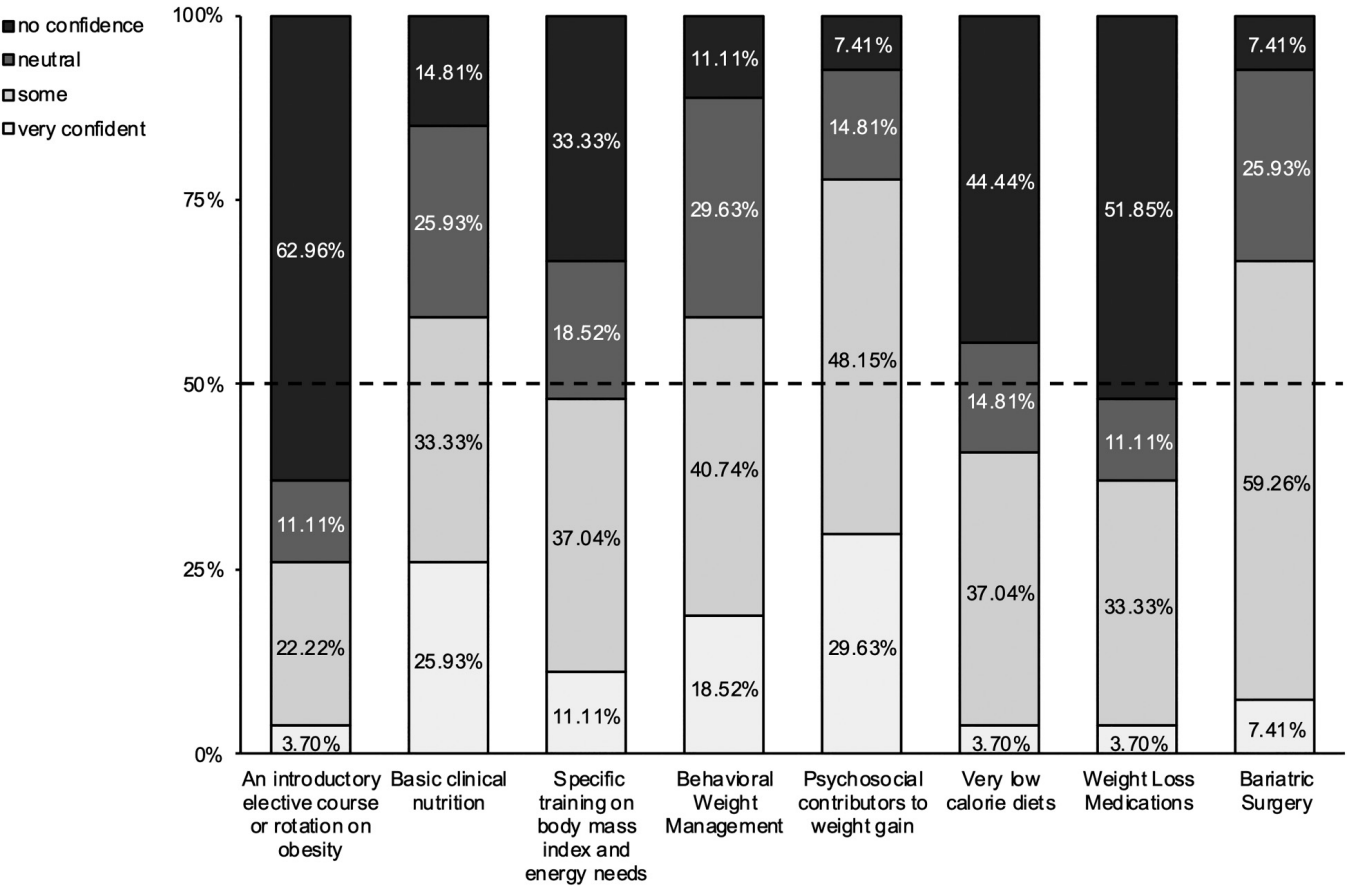
Faculty Confidence to Teach Adult Obesity.

Faculty (n = 27) rated their confidence to teach adult obesity in eight areas: an introductory course on obesity; basic nutrition; BMI and energy needs;, behavioral weight management; psychosocial contributors to weight gain; very low-calorie diets; weight loss medications; and bariatric surgery (Figure 2). Four out of eight items reached the 50% threshold for some confidence or very confident: basic nutrition, behavioral weight loss management, psychosocial contributors to weight gain, and bariatric surgery. Four out of eight items were neutral or no confidence: an introductory course on obesity, BMI and energy needs, very low-calorie diets, and weight loss medications.

Faculty response rates for the question “*Q4.2 Do you feel you have the required confidence to teach residents/students about adult obesity?”* are plotted as percentage of respondents for each of the 8 teaching confidence categories assessed: 1) an introductory course on obesity, 2) basic nutrition, 3) BMI and energy needs, 4) behavioral weight management, 5) psychosocial contributors to weight gain, 6) very low-calorie diets, 7) weight loss medications and 8) bariatric surgery. A stacked plot is used to show the total percentage of responses out of 100% for “very confident” (white), “some confidence” (light gray), “neutral” (dark gray), or “no confidence” (black). The dotted line indicates the 50% threshold of response rates.

#### Importance of interprofessional collaboration

Faculty (n = 27) were asked to rate the importance of interprofessional collaboration on the development of obesity curriculum. Most respondents indicated that they felt it was “very important” (37.04%, n = 10/27) and “extremely important” (37.04%, n = 10/27) to have interprofessional collaboration; 22.22% were neutral (n = 6/27) and 3.70% (1/27) felt it was slightly important.

#### Confidence to treat

**Figure 3.**
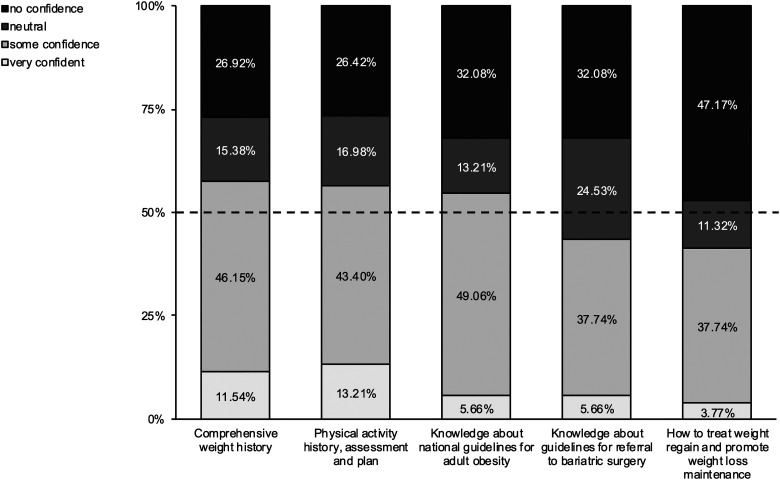
Confidence to Treat Adult Obesity.

Faculty, Residents, and Students (n = 52) rated their confidence to treat adult obesity in five areas: weight history; physical activity history; knowledge of national guidelines for adult obesity; knowledge of national guidelines for bariatric surgery; and how to treat weight gain and promote weight loss maintenance (Figure 3). Three out of five items reached the 50% threshold for some confidence or very confident for: weight history, physical activity history, and knowledge of national guidelines for adult obesity. Two out of five items were neutral or no confidence; knowledge about national guidelines for bariatric surgery, and how to treat weight gain and promote weight loss maintenance.

Faculty, student and resident response rates for the question *“Q6.2 Do you feel you have the required confidence to treat adult obese patients?”* are plotted as percentage of respondents for each of the 5 treatment confidence categories assessed: 1) comprehensive weight history, 2) physical activity, history assessment and plan, 3) knowledge about national guidelines for adult obesity, 4) knowledge about guidelines for referral to bariatric surgery, and 5) how to treat weight gain and promote weight loss maintenance. A stacked plot is used to show the total percentage of responses out of 100% for “very confident” (white), “some confidence” (light gray), “neutral” (dark gray), or “no confidence” (black). The dotted line indicates the 50% threshold of response rates.

#### Faculty versus trainee confidence to treat

Using Fisher exact analyses with a 2-tailed α of .05, there were no significant differences found between faculty and student responses on all five areas queried related to confidence to treat: weight history (*p* = .69); physical activity history (*p* = .17); knowledge of national guidelines for adult obesity (*p* = .21); knowledge of national guidelines for bariatric surgery (*p* = .46); and how to treat weight gain and promote weight loss maintenance (*p* = .46).

#### Clinic site rotations

Eighty percent (24/30) of the trainees did not attend any clinic site rotations on obesity. The researchers do not know if any clinic site rotations were available or offered to the respondents.

#### Support for centralized obesity treatment center

Faculty, Students, and Residents (n = 52) rated whether they believed the institution could benefit from a centralized adult obesity treatment center. Most respondents (78.85%) “strongly agreed” (55.77%, n = 29/52) and “agreed” (23.08%, n = 12/52) that the institution could benefit from a centralized obesity treatment center. There were no significant differences between faculty and student/resident responses (Fisher exact test, *p* = .37).

#### Need for “Obesity 101” introductory type course

Faculty, Students, and Residents (n = 55) rated whether they thought primary health care trainees need an “Obesity 101” course. Most respondents said “yes” (80%, n = 44/55). (Obesity 101 course was used in the general sense of an introductory course on obesity).

#### Ranking of needed topics for obesity training

Faculty (n = 26) were asked to rank the order of importance of adult obesity training topics from 1 (most important) to 9 (least important) (Table 2). In Table 2, the highest percentage of respondents for a given rank is highlighted for each topic. The three highest ranked topics were: basic nutrition, behavioral weight management, and a rotation on adult obesity.

**Table 2.** Ranking of Topics Needed for Obesity Training.

	RANKING								
TOPIC	1	2	3	4	5	6	7	8	9
Basic nutrition	34.62%	15.38%	7.69%	15.38%	3.85%	11.54%	7.69%	3.85%	0.00%
Behavioral weight management	26.92%	15.38%	23.08%	26.92%	7.69%	0.00%	0.00%	0.00%	0.00%
A rotation on adult obesity	23.08%	3.85%	7.69%	3.85%	15.38%	0.00%	7.69%	7.69%	30.77%
An introductory elective course on adult obesity	3.85%	30.77%	7.69%	3.85%	7.69%	15.38%	7.69%	15.38%	7.69%
Psychosocial contributors to weight gain	0.00%	19.23%	23.08%	19.23%	11.54%	3.85%	7.69%	7.69%	7.69%
Specific training on BMI and energy needs	3.85%	7.69%	15.38%	7.69%	15.38%	23.08%	3.85%	11.54%	11.54%
Weight loss medications	3.85%	0.00%	3.85%	19.23%	11.54%	30.77%	19.23%	3.85%	7.69%
Bariatric surgery	3.85%	3.85%	7.69%	0.00%	15.38%	7.69%	34.62%	15.38%	11.54%
Very low-calorie diets	0.00%	3.85%	3.85%	3.85%	11.54%	7.69%	11.54%	34.62%	23.08%

Abbreviation: BMI, body mass index.

#### Use of outside weight management services

Faculty, students, and residents were asked whether they used weight management services for their patients outside of our institution's system (yes, no, not applicable). Over 50% [56.6% (30/53)] of respondents stated “no” they did not use weight management services outside of the institution's services and 22.6% (12/53). Of those who responded “yes” [22.6% (12/53)] most said they used Weight Watchers. One respondent used My Fitness Pal, Inc and one used a local weight management program. One respondent used Jenny Craig. One other respondent mentioned Apple Health and Fitbit. Of those respondents who responded “no” [43.40%(23/53)], said the primary reason was that they were not aware of other weight management services or not familiar with them. One respondent stated that “I’ve had no training on what services outside [institution's] are available.” Eleven out of 53 responded “not applicable.”

#### Comments from survey respondents

Only two respondents answered the open text box request for comments.

### First Comment

“Many patients need obesity care, and it would be good to have ‘HIGH QUALITY’ referral options (dietician support, bariatric services, counseling) that were covered by insurance and did not refuse referrals and coordinated by primary care. The issue is not typically lack of provider knowledge, it is lack of covered resources for patients at [institution], lack of time in the primary care setting, and lack of patient ability to follow the plan given their other health and life needs. I do not want obesity care removed from primary care as it should remain integrated with other primary care (hypertension, depression, diabetes, social needs). Primary care just needs more support. A better provider understanding of what each option entails, costs, scheduling time frame, would be helpful.”

### Second Comment

Behavioral health integration and support (especially for publicly insured patients) is “INADEQUATE.”

#### Key Informant Interviews Results (please see Supplement 2 for a complete list of questions)

Thematic analysis (Supplement 3) of four key informant Administrator respondents revealed that adult obesity was often covered, in their curricula, in the context of its comorbidities such as metabolic syndrome, diabetes, and heart disease, rather than as a disease itself. In general, they reported that although students and residents received clinical exposure to people with adult obesity, they were not aware of any formal clinical rotations on adult obesity that students could take. In terms of barriers to care, all Administrators stated that there were some topics on adult obesity being offered but were not aware of any formal obesity curriculum for FNP/PA students and/or for FM/IM residents. All Administrators stated that there was not enough time in the curriculum to teach adult obesity; however, all felt that there was a need to integrate the topic into other related training subjects and offer students the ability to get more experience in such topics as motivational interviewing, weight management, antiobesity drugs, and bariatric surgery. They also expressed a need for more dedicated content on adult obesity. Financial limitations were also brought up as a barrier, not only in the context of adequate revenues and staffing to expand the curriculum but also, in terms of health insurance coverage for weight management services, such as weight loss classes, very low-calorie diets, and antiobesity drugs. In addition, they supported the need for institutional coordinated adult obesity treatment.

Administrators were also asked about opportunities for improvement. Certainly, all felt that their curricula and training programs could improve course content and clinical experience in adult obesity, and they were willing to consider addressing the topic of adult obesity in their teaching, curricula. They also felt that there was a need to add more content on lifestyle medicine, behavioral health, nutrition, physical activity, in addition to more instruction on the pharmacological management of obesity and bariatric surgery guidelines.

Regarding credentialing exams, Administrators stated that they would need to examine existing requirements further to ascertain exam requirements on the topic of adult obesity.

In terms of clinical treatment services and additional recommendations: Administrators were not aware of a formal obesity treatment center and were not always certain as to where providers could refer patients with obesity for weight management. Administrators also had a wealth of recommendations including some of the following: appoint a point person on faculty as an expert on obesity who can develop the curriculum; have more dedicated content on obesity; teach more on the etiology of obesity, nutrition, energy metabolism, and genetic components; develop an online module on obesity; develop short online modules; offer a clinical rotation on obesity; rotate with a registered dietitian; and provide opportunities for a clinical rotation in the bariatric surgery department.

### Quotes From the Key Administrators

“They [FNP/PA students] are getting more teaching on the [comorbidities of obesity] disease process than on obesity itself.”

“My sense is that we say: “maintain healthy weight,” but we don’t necessarily dive into how to do that.”

“Barriers that we would experience would be how to find the time to cover it all.”

“We need to look at our certifying exams and what requirements are for certification exams and then be able to augment the curriculum to cover lifestyle topics.”

“The issue we had with the weight management clinic is that there is not enough revenue.”

Please see Supplement 3 for Administrator interview theme tables.

## Discussion

Obesity is a complex chronic disease and is associated with serious health risks, which can lead to increased morbidity and mortality.^22,23^ Primary care providers play a critical role in supporting and offering nonbiased treatment to people who are obese.^24–26^ Obesity treatment modalities include, but are not limited to nutrition, physical activity, behavioral health, pharmacotherapy, and bariatric surgery. It is crucial that healthcare providers who treat people who are obese not only understand the disease process but also provide interprofessional collaboration and case management services.^23,25–27^

Although research on the effectiveness of medical education trainees’ competencies about obesity is still limited there have been significant findings in several areas, including medical residencies, nursing training, PA programs, and credentialing exams. Some of these studies have demonstrated that implementing obesity and nutrition-related curriculum can improve trainees’ knowledge.^5,15,18,28,29^ Some studies support the need in family and internal medicine programs for a multicomponent approach to address obesity curriculum development including clinical nutrition; physical activity; psychosocial aspects; and pharmacologic and surgical treatment modalities.^2,6,14,28,30,31^

Research by Butsch et al concluded that the surveyed IM residency programs did not adequately cover such topics as etiological aspects, psychological aspects, pharmacological treatment, and weight stigma of obesity.^6^ Furthermore, the authors point out that “lack of room” and faculty expertise were the greatest barriers to integrating obesity education into their curricula.^6^ In addition, Ferrante et al found family medicine doctors lack adequate preparation to recommend proper treatment for patients with extreme obesity, such as bariatric surgery and prescribing antiobesity drugs.^32^

Limited studies surveying NP and PA schools also report the need for evidence-based information about obesity drugs, diet, and exercise; that there are limited obesity-specific curricula; and that there is a need for further training.^4,5,13,15^

This leads us to a discussion about how US medical, nursing and PA programs match obesity education with credentialing exams. A study by Kushner et al, looking at US Medical Licensing Examination (USMLE) exam questions related to obesity, found that most questions related to the comorbidities of obesity rather than the evaluation of USMLE's students’ knowledge about the diagnosis and management of obesity itself. Their review concluded that there is insufficient coverage of obesity in the USMLE Step examinations.^33^ The USMLE can look for areas of improvement on testing about clinical obesity, in addition to testing on obesity-related comorbidities.^34^ Our key informant interviews support these concerns.

Several studies show promise with targeted training programs. They concluded that more research is also needed to evaluate training pre- and posttest knowledge on obesity-related topics.^29,35–37^

This innovative pilot study supports previous research about medical training on obesity and looks at opportunities for improvement in medical education, curricular development, and treatment services on adult obesity at our institution. It examined the opinions of faculty and trainees from our institution's IM, FM, NP, and PA training programs. Our study is novel in that we developed an original survey and key informant interviews, which asked questions related to student and faculty opinions, experiences, and recommendations about their curriculum and clinical training on adult obesity. Furthermore, our survey asked questions related to interprofessional care, examining the specific competencies for adult obesity care, such as lifestyle, behavioral health, antiobesity drugs, and bariatric surgery. It revealed that the three highest ranked topics were basic nutrition, behavioral weight management, and a rotation on adult obesity. Residents and students strongly supported an introductory type “Obesity 101” course. Faculty, residents, and students strongly supported a centralized obesity treatment center and more interprofessional collaboration. These findings support the importance of training with multiple professionals who treat people who are obese as well as the need for a treatment center where trainees can gain exposure to experts in the field and their clients. Our key informant Administrator interviews revealed the need for more expert faculty and training on adult obesity. They identified the need for more fiscal resources and time limitations in an already crowded curriculum. They recommended the development of online teaching modules about adult obesity and offering clinical exposure to weight management. Online learning has been evolving and offers a concise way to develop training modules and videos for student learning. These hybrid models may lend themselves well to an already crowded curriculum.

A synthesis of information by Eliot et al concluded that there is also limited training for healthcare professionals on how to collaborate with other members of obesity treatment teams, which may lead to improved shared treatment decisions.^17^

Our study in conjunction with the cited research studies, support the need for additional training on obesity for primary care trainees and that adding additional curriculum on obesity can improve their knowledge. They also provide additional justification for our hypotheses and study results that our institution's IM/FM residents; and SON FNP/PA students need additional training in the clinical assessment and treatment of adult obesity; and more exposure to interprofessional training.

Our results supported the hypotheses that primary care faculty, residents, and students would indicate the need for additional training in the clinical assessment and treatment of adult people with obesity and that adult obesity treatment services can be improved. They also supported the hypothesis that additional interprofessional services and collaboration are needed. This study reinforces previous research on adult obesity training in medical residency, nursing, physician assistant programs, and medical schools. It further reinforces previous research studies that recommend that primary healthcare providers should have competencies in nutrition, physical activity, and behavioral medicine; an understanding of very low-calorie diets, antiobesity drugs and when to refer to bariatric surgery. In addition, treating patients with obesity requires a case management approach and interprofessional collaboration. Our study also revealed that barriers to training include the need for more dedicated time in training programs, school finances, and having faculty resources.

Our results also provide new insights on faculty and student perceptions about how their education and training can be improved. They also show that there is promising evidence that primary care nursing, physician assistant, and medical residency programs can look for new and innovative ways to increase trainees’ knowledge about some of the elements of lifestyle medicine; obesity assessment and treatment; and interprofessional care.

### Limitations

This study was a pilot study and as such had limited funding. It had a limited sample size for the survey and had a small group of key informants. A power analysis of the sample size was not performed because this was a sample of convenience. With a greater sample size, it is possible that some of the results, which were not statistically significant, may have become significant and more generalizable. The single recruitment site and the limited sample size limit the generalizability of our results. Nonetheless, they provide key insight into providers obesity training/education, and these results can inform future studies.

Also, this survey was conducted at only one UC academic institution and although the results may be generalizable to other institutions, this remains to be determined. Also, this survey did not include hypotheses or objectives to evaluate areas related to weight stigma.

This survey and set of interviews asking respondents about their opinions was not validated. Although the survey had over 70 respondents, respondents were primarily from the IM Residency program with few respondents from SON and the FM Residency programs. This pilot study did not have the resources to review in detail either FM/IM or FNP/PA curriculum on adult obesity, but rather relied on the key informant interviews and survey questions to ascertain general content areas and recommendations. This study did not examine race/ethnic variables.

### Future Directions

Further research and curriculum development are necessary in the field of obesity education. This research design can be built upon and expanded to other health care universities. The pilot survey could be refined by adding additional questions and validating them. Questions could be developed to examine whether there are any race/ethnic disparities or weight bias in medical training programs, and how to achieve health equity in the assessment and treatment of people who are obese. In addition, further refinement of key informant questions and interviewer interrater reliability could be improved. In addition, student-centered and hybrid (remote and in person) learning approaches need to be explored further. This can lead the way to improved obesity training programs with the goal of preventing future obesity-related morbidity and mortality. Research populations could be expanded to include historically Black colleges and universities and other at-risk populations, such as pediatrics, obstetrics/gynecology, and endocrinology.

It is important for us to understand the root causes of unhealthy weight. In addition to environmental, lifestyle, and genetic contributors, we must also strive to understand how we educate our healthcare providers to support people with obesity to achieve a healthy weight.

## Conclusions

Primary care faculty, IM/FM residents, and FNP/PA students at our institution see the need for more education and clinical training on adult obesity. Trainees need more education on such topics as nutrition, physical activity, behavioral health, antiobesity medications, and bariatric surgery. They also need more confidence treating excess weight gain and supporting weight loss maintenance. Survey respondents also felt that adult obesity treatment programs may benefit from a centralized obesity treatment center and improved interprofessional collaboration. Administrators felt that primary care training programs can build upon current curricula and clinical rotations as well as look for innovative ways, such as hybrid learning, to improve education on adult obesity. Medical education programs can look for opportunities to incorporate additional curricula on obesity and consider clinical rotations that provide care to people who are obese. More research is needed on adult obesity medical education and how this training is aligned with licensing exams.

## Supplemental Material

sj-docx-1-mde-10.1177_23821205241269371 - Supplemental material for Perceptions of Adult Obesity Education: A Pilot Study Supplemental material, sj-docx-1-mde-10.1177_23821205241269371 for Perceptions of Adult Obesity Education: A Pilot Study by Seleda Ann Williams, Cara Marie Sandholdt, Jeffrey Robert Fine and Kougang Anne Mbe in Journal of Medical Education and Curricular Development

sj-docx-2-mde-10.1177_23821205241269371 - Supplemental material for Perceptions of Adult Obesity Education: A Pilot Study Supplemental material, sj-docx-2-mde-10.1177_23821205241269371 for Perceptions of Adult Obesity Education: A Pilot Study by Seleda Ann Williams, Cara Marie Sandholdt, Jeffrey Robert Fine and Kougang Anne Mbe in Journal of Medical Education and Curricular Development

sj-docx-3-mde-10.1177_23821205241269371 - Supplemental material for Perceptions of Adult Obesity Education: A Pilot Study Supplemental material, sj-docx-3-mde-10.1177_23821205241269371 for Perceptions of Adult Obesity Education: A Pilot Study by Seleda Ann Williams, Cara Marie Sandholdt, Jeffrey Robert Fine and Kougang Anne Mbe in Journal of Medical Education and Curricular Development

sj-docx-4-mde-10.1177_23821205241269371 - Supplemental material for Perceptions of Adult Obesity Education: A Pilot Study Supplemental material, sj-docx-4-mde-10.1177_23821205241269371 for Perceptions of Adult Obesity Education: A Pilot Study by Seleda Ann Williams, Cara Marie Sandholdt, Jeffrey Robert Fine and Kougang Anne Mbe in Journal of Medical Education and Curricular Development

sj-docx-5-mde-10.1177_23821205241269371 - Supplemental material for Perceptions of Adult Obesity Education: A Pilot Study Supplemental material, sj-docx-5-mde-10.1177_23821205241269371 for Perceptions of Adult Obesity Education: A Pilot Study by Seleda Ann Williams, Cara Marie Sandholdt, Jeffrey Robert Fine and Kougang Anne Mbe in Journal of Medical Education and Curricular Development
