# Supplementary material for: Perceptions of Adult Obesity Education: A Pilot Study
Source: J Med Educ Curric Dev. 2024 Oct 1;11:23821205241269371. doi: 10.1177/23821205241269371 (PMC11450567; doi:10.1177/23821205241269371)
Supplement: sj-docx-1-mde-10.1177_23821205241269371 - Supplemental material for Perceptions of Adult Obesity Education: A Pilot Study [file sj-docx-1-mde-10.1177_23821205241269371.docx]

**University of California (UCD) Adult Obesity Primary Care Survey DRAFT NOT FOR DISTRIBUTION**

**Survey Flow**

**Standard: Consent (1 Question) Standard: Demographics (1 Question) Standard: Trainee Only (6 Questions) Standard: All Faculty (4 Questions)**

**Standard: IM/FM or SON Faculty Only (2 Questions) Block: All (11 Questions)**

Page Break

**Please also see link inserted into HRP 501 Research Protocol, Section 7 Protocols Involved - Page 9 out of 16 :**

**Link to Qualtrics Adult Obesity Primary Care Survey**:

[**https://ucdavis.co1.qualtrics.com/jfe/form/SV_0xErO8eBN5qQgLz**](https://ucdavis.co1.qualtrics.com/jfe/form/SV_0xErO8eBN5qQgLz)

**Proceed to next page for Word version of survey**

# Start of Block: Consent

Q1.1

ADULT OBESITY PRIMARY CARE SURVEY ABOUT THIS SURVEY:

We would like to get your opinions about UCD adult obesity and weight management training of primary care residents, nursing students, and physician assistant students; and about UCD outpatient adult treatment services. This survey is targeted to UCD Internal Medicine (IM) and Family and Community Medicine (FM) faculty and PGY 3 residents as well as UCD Betty Irene School of Nursing (SON) 2nd Year Family Nurse Practitioner (FNP) students and 2nd Year UCD Physician Assistant students.

Some of the questions are for UCD IM/FM/SON faculty only and some of the questions are for UCD IM/FM residents/SON FNP//PA students only. Some of the questions are for both faculty and residents/students. This survey is designed to take you to these questions after you check your department and staff type.

Your opinions and advice are very important. The results of this survey will be used to generate recommendations to UCD administration on whether and how to improve obesity training and outpatient services and publish these results in a journal such as

MedEdPortal.

This survey is anonymous. Summary results will be deidentified and steps will be taken to keep all personal information confidential. This survey has been approved by the UCD IRB (pending approval). This survey will take about 20 minutes of your time to

complete.

For any questions or concerns about this survey you can contact Dr. Seleda Williams at [drswilliams@ucdavis.edu.](mailto:drswilliams@ucdavis.edu) Please take time to read the attached consent form. [Consent form for obesity survey](https://ucdavis.co1.qualtrics.com/CP/File.php?F=F_5vdSu6yrrFsyq6V)

Next, please click "YES" to consent to this survey or "NO" to decline this survey.

o YES (1)

o NO (2)

*Skip To: End of Block If ADULT OBESITY PRIMARY CARE SURVEY ABOUT THIS SURVEY: We would*

*like to get your opinions about UCD... = YES*

*Skip To: End of Survey If ADULT OBESITY PRIMARY CARE SURVEY ABOUT THIS SURVEY: We*

*would like to get your opinions about UCD... = NO*

# End of Block: Consent

**Start of Block: Demographics**

BLANK PAGE

Q2.1

UCD POSITION:

Please check your UCD Department Program and whether you are faculty, resident or nursing student or physician assistant student.

o IM Faculty (1)

o IM Resident PGY 3 (2)

o FM Faculty (3)

o FM Resident (PGY 3) (4)

o SON Faculty (5)

o SON FNP student (2nd Year) (6)

o SON PA student (2nd Year) (7)

o Other (8)

*Skip To: End of Survey If UCD POSITION: Please check your UCD Department Program and whether you are faculty, resident or n... = Other*

# End of Block: Demographics

**Start of Block: Trainee Only**

*Display This Question:*

*If UCD POSITION: Please check your UCD Department Program and whether you are faculty, resident or n... = IM Resident PGY 3*

*Or UCD POSITION: Please check your UCD Department Program and whether you are faculty, resident or n... = FM Resident (PGY 3)*

*Or UCD POSITION: Please check your UCD Department Program and whether you are faculty, resident or n... = SON FNP student (2nd Year)*

*Or UCD POSITION: Please check your UCD Department Program and whether you are faculty, resident or n... = SON PA student (2nd Year)*

Q3.1

FOR RESIDENTS & STUDENTS ONLY QUESTIONS : NEXT 7 QUESTIONS

For purposes of this survey obesity training will be defined as any lectures or rotations you have had related to the following topics: basic clinical nutrition, physical activity counseling, behavioral modification, psychosocial issues related to adult obesity, adult obesity diagnosis and treatment.

During your UCD IM/FM residency, or SON FNP/PA program how much training did you receive in each of the following areas? Please select appropriate level of training for each area.

| Extensive (full course or rotation) (1) | | Moderate (part of a course or rotation) (2) | Limited (1 or less lecture or rotation talk) (3) | No Training (4) |
| --- | --- | --- | --- | --- |
| obesity epidemiology (1) | o | o | o | o |
| basic clinical nutrition (2) | o | o | o | o |
| physical activity guidelines (3) | o | o | o | o |
| behavioral weight loss strategies (4) | o | o | o | o |
| psychosocial contributors to weight gain and obesity (5) | o | o | o | o |
| how to diagnose & treat obesity (6) | o | o | o | o |
| weight loss medications (7) | o | o | o | o |
| bariatric surgery (8) | o | o | o | o |

*Display This Question:*

*If UCD POSITION: Please check your UCD Department Program and whether you are faculty, resident or n... = IM Resident PGY 3*

*Or UCD POSITION: Please check your UCD Department Program and whether you are faculty, resident or n... = FM Resident (PGY 3)*

*Or UCD POSITION: Please check your UCD Department Program and whether you are faculty, resident or n... = SON FNP student (2nd Year)*

*Or UCD POSITION: Please check your UCD Department Program and whether you are faculty, resident or n... = SON PA student (2nd Year)*

lectures or teaching modules on weight management for adult obese patients?

o Yes (1)

o No (2)

o Not sure (3)

*Display This Question:*

*If UCD POSITION: Please check your UCD Department Program and whether you are faculty, resident or n... = IM Resident PGY 3*

*Or UCD POSITION: Please check your UCD Department Program and whether you are faculty, resident or n... = FM Resident (PGY 3)*

*Or UCD POSITION: Please check your UCD Department Program and whether you are faculty, resident or n... = SON FNP student (2nd Year)*

*Or UCD POSITION: Please check your UCD Department Program and whether you are faculty, resident or n... = SON PA student (2nd Year)*

Q3.3 If you answered YES to Question **#4**, please indicate what type of lectures:

*Display This Question:*

*If UCD POSITION: Please check your UCD Department Program and whether you are faculty, resident or n... = IM Resident PGY 3*

*Or UCD POSITION: Please check your UCD Department Program and whether you are faculty, resident or n... = FM Resident (PGY 3)*

*Or UCD POSITION: Please check your UCD Department Program and whether you are faculty, resident or n... = SON FNP student (2nd Year)*

*Or UCD POSITION: Please check your UCD Department Program and whether you are faculty, resident or n... = SON PA student (2nd Year)*

rotations on adult obesity and weight management? Please check which one. If yes, please also indicate approximate length of the clinic rotation.

o One or more months (1)

o Less than one month (2)

o One week or less (3)

o One day or less (4)

o None (5)

o Not sure (6)

o Not applicable (7)

*Display This Question:*

*If UCD POSITION: Please check your UCD Department Program and whether you are faculty, resident or n... = IM Resident PGY 3*

*Or UCD POSITION: Please check your UCD Department Program and whether you are faculty, resident or n... = FM Resident (PGY 3)*

*Or UCD POSITION: Please check your UCD Department Program and whether you are faculty, resident or n... = SON FNP student (2nd Year)*

*Or UCD POSITION: Please check your UCD Department Program and whether you are faculty, resident or n... = SON PA student (2nd Year)*

SON FNP/PA program:

| training in this area is adequate (1) | | no additional training is needed (2) | some additional training is needed (3) | much more training is needed (4) |
| --- | --- | --- | --- | --- |
| Obesity epidemiology (1) | o | o | o | o |
| Basic clinical nutrition (2) | o | o | o | o |
| Physical activity guidelines (3) | o | o | o | o |
| Behavioral weight loss strategies (4) | o | o | o | o |
| Psychosocial contributors to weight gain and obesity (5) | o | o | o | o |
| How to diagnose and treat adult obesity (6) | o | o | o | o |
| Weight loss medications (7) | o | o | o | o |
| Bariatric surgery (8) | o | o | o | o |

*Display This Question:*

*If UCD POSITION: Please check your UCD Department Program and whether you are faculty, resident or n... = IM Resident PGY 3*

*Or UCD POSITION: Please check your UCD Department Program and whether you are faculty, resident or n... = FM Resident (PGY 3)*

*Or UCD POSITION: Please check your UCD Department Program and whether you are faculty, resident or n... = SON FNP student (2nd Year)*

*Or UCD POSITION: Please check your UCD Department Program and whether you are faculty, resident or n... = SON PA student (2nd Year)*

course?

o Yes (1)

o Not Sure (2)

o Neutral (3)

o No (4)

# End of Block: Trainee Only

**Start of Block: All Faculty**

*Display This Question:*

*If UCD POSITION: Please check your UCD Department Program and whether you are faculty, resident or n... = IM Faculty*

*Or UCD POSITION: Please check your UCD Department Program and whether you are faculty, resident or n... = FM Faculty*

*Or UCD POSITION: Please check your UCD Department Program and whether you are faculty, resident or n... = SON Faculty*

Q4.1

FOR ALL UCD FACULTY ONLY

Please rank the importance of adult obesity training for residents and students.

# Please drag and drop in order of importance from 1-9.

Basic nutrition (1)

Specific training on body mass index and energy needs (2)

Behavioral weight management (3)

Weight loss medications (4)

Bariatric surgery (5)

Very low- calorie diets (6)

Psychosocial contributors to weight gain (7)

An introductory elective course on adult obesity (8)

A rotation on adult obesity (9)

Q4.2 Do you feel you have the required confidence to teach residents/students about adult obesity?

| no confidence (1) | | some confidence (2) | neutral (3) | very confident (4) |
| --- | --- | --- | --- | --- |
| Basic nutrition (1) | o | o | o | o |
| Specific training on body mass index and energy needs (2) | o | o | o | o |
| Behavioral weight management (3) | o | o | o | o |
| Weight loss medications (4) | o | o | o | o |
| Bariatric surgery (5) | o | o | o | o |
| Very low- calorie diets (6) | o | o | o | o |
| Psychosocial contributors to weight gain (7) | o | o | o | o |
| An introductory elective course or rotation on obesity (8) | o | o | o | o |

Q4.3 Do you think UCD primary care health care provider trainees need a “Obesity 101” type course?

o Yes (1)

o Not Sure (2)

o Neutral (3)

o No (4)

*Display This Question:*

*If UCD POSITION: Please check your UCD Department Program and whether you are faculty, resident or n... = IM Faculty*

*Or UCD POSITION: Please check your UCD Department Program and whether you are faculty, resident or n... = FM Faculty*

*Or UCD POSITION: Please check your UCD Department Program and whether you are faculty, resident or n... = SON Faculty*

Q4.4 How important is it for you to have more interprofessional collaboration regarding obesity curriculum development?

o Extremely important (1)

o Very important (2)

o Neutral (3)

o Moderately important (4)

o Slightly important (5)

o Not at all important (6)

# End of Block: All Faculty

**Start of Block: IM/FM or SON Faculty Only**

*Display This Question:*

*If UCD POSITION: Please check your UCD Department Program and whether you are faculty, resident or n... = IM Faculty*

*Or UCD POSITION: Please check your UCD Department Program and whether you are faculty, resident or n... = FM Faculty*

Q5.1

FOR IM/FM FACULTY ONLY

Do you think UCD IM/FM residents have the required training in adult obesity to successfully answer a board exam question on this topic?

| extensive training (e.g.: full course or rotation) (1) | | moderate training (e.g.: topics discussed as part of another course or rotation (2) | very little training (e.g.: less than or equal to 1 lecture) (3) | no training (4) |
| --- | --- | --- | --- | --- |
| Obesity epidemiology (1) | o | o | o | o |
| Basic clinical nutrition (2) | o | o | o | o |
| Physical activity guidelines (3) | o | o | o | o |
| Behavioral weight loss strategies (4) | o | o | o | o |
| Psychosocial contributors to weight gain and obesity (5) | o | o | o | o |
| How to diagnose and treat adult obesity (6) | o | o | o | o |
| Weight loss medications (7) | o | o | o | o |
| Bariatric surgery (8) | o | o | o | o |

*Display This Question:*

*If UCD POSITION: Please check your UCD Department Program and whether you are faculty, resident or n... = SON Faculty*

Q5.2

FOR SON FNP/PA FACULTY ONLY

Do you think UCD FNP/PA students have the required training in adult obesity to meet any FNP/PA certification exam questions on this topic?

| extensive training (e.g.: full course or rotation) (1) | | moderate training (e.g.: topics discussed as part of another course or rotation (2) | very little training (e.g.: less than or equal to 1 lecture) (3) | no training (4) |
| --- | --- | --- | --- | --- |
| Obesity epidemiology (1) | o | o | o | o |
| Basic clinical nutrition (2) | o | o | o | o |
| Physical activity guidelines (3) | o | o | o | o |
| Behavioral weight loss strategies (4) | o | o | o | o |
| Psychosocial contributors to weight gain and obesity (5) | o | o | o | o |
| How to diagnose and treat adult obesity (6) | o | o | o | o |
| Weight loss medications (7) | o | o | o | o |
| Bariatric surgery (8) | o | o | o | o |

# End of Block: IM/FM or SON Faculty Only

**Start of Block: All**

Q6.1

REMAINING QUESTIONS FOR ALL FACULTY AND STUDENTS

In your estimate, what percent of UCD adult obese patients would benefit from weight loss for medical reasons? Medical reasons include such reasons as the need to reduce obesity co- morbidities such as hypertension, dyslipidemias, congestive heart failure, obstructive sleep apnea, joint arthropathies, etc. Depending on the level of obesity, reasons might also include need to lose weight for bariatric or cardiac surgery, etc.

o 0-20% (1)

o 21-40% (2)

o 41-60% (3)

o 61-80% (4)

o 81-100% (5)

o I cannot estimate (6)

| no confidence (1) | | some confidence (2) | neutral (3) | very confident (4) |
| --- | --- | --- | --- | --- |
| Conducting a comprehensive weight history that would include such items as; weights through the life phases; past attempts at weight loss; physical activity levels; and 24- hour dietary recall. (1) | o | o | o | o |
| Conducting a physical activity history assessment and plan, using national guidelines for physical activity (2) | o | o | o | o |
| Knowledge about national guidelines for the assessment and treatment of adult obesity (3) | o | o | o | o |
| Knowledge about guidelines for referral to bariatric surgery programs (4) | o | o | o | o |
| Knowledge about how to treat weight regain and promote weight loss maintenance (5) | o | o | o | o |

|  | rarely (1) | sometimes (2) | often (3) |
| --- | --- | --- | --- |
| Healthy lifestyle classes (8) | o | o | o |
| Nutrition and diet consultation with a registered dietitian (1) | o | o | o |
| Physical therapy and/or pool therapy (2) | o | o | o |
| Behavioral health (3) | o | o | o |
| UCD Nutrition Clinic (4) | o | o | o |
| Very low-calorie diet program (5) | o | o | o |
| Bariatric surgery (6) | o | o | o |
| Not applicable, I do not treat any obese patients (7) | o | o | o |

adults?

| no services are available (1) | | some services are available, but more are needed (2) | some services are available and adequate to meet needs (3) | plenty of services are available (4) | no others services are needed (5) |
| --- | --- | --- | --- | --- | --- |
| Healthy Lifestyle Classes (8) | o | o | o | o | o |
| Nutrition and diet consultation with a registered dietitian (1) | o | o | o | o | o |
| Physical therapy and/or pool therapy (2) | o | o | o | o | o |
| Behavioral health (3) | o | o | o | o | o |
| Nutrition Clinic (4) | o | o | o | o | o |
| Very low- calorie diet program (5) | o | o | o | o | o |
| Bariatric surgery (6) | o | o | o | o | o |
| Not applicable, I do not treat any obese patients (7) | o | o | o | o | o |

such as Weight Watchers, or very low-calorie diet programs?

o Yes (1)

o No (2)

o Not Applicable (3)

*Skip To: Q6.6 If Do you use weight management services for your adult obese patients outside of UCD, such as Weigh... = Yes*

*Skip To: Q6.7 If Do you use weight management services for your adult obese patients outside of UCD, such as Weigh... = No*

*Skip To: Q6.8 If Do you use weight management services for your adult obese patients outside of UCD, such as Weigh... = Not Applicable*

Q6.6

If you answered yes to Question #6.5, please indicate which outside weight management services you referred your obese patient to?

Q6.7 If you answered “No” to Question #6.5, please discuss why you did not use outside weight management services?

▢ referral availability to an expert in weight loss medications. (1)

▢ care coordination for obese patients. (2)

▢ training of IM/FM residents and SON FNP/PA on adult obesity (3)

▢ training of UCD faculty on adult obesity treatment. (4)

▢ administrative support for obesity training and treatment services for UCD faculty. (5)

▢ more funding for obesity training at UCD. (6)

▢ more funding for obesity treatment services at UCD (7)

programs? Please select all that apply.

▢ Patients have inadequate or no health insurance coverage for weight management classes. (1)

▢ I am not sure how to bill for weight management classes. (2)

▢ Patients have inadequate or no health insurance coverage for weight loss medications (3)

▢ I am not sure how to bill for weight loss medications. (4)

▢ Reluctance on the part of my patients to participate in weight loss programs (5)

▢ Lack of obesity treatment referral services (6)

▢ I am too busy to treat obese patients in my practice (7)

▢ Not applicable (EXCLUDE FROM DATA COLLECTION) (8)

▢ Other: INSERT TEXT BOX (9)

other words a UCD department or an office for coordination of clinical services for obesity treatment and referrals.

o Strongly agree (1)

o Agree (2)

o Somewhat agree (3)

o Neither agree nor disagree (4)

o Somewhat disagree (5)

o Disagree (6)

o Strongly disagree (7)

Q6.11 Please list any other concerns or recommendations regarding adult obesity training and/or treatment services at UCD.

# End of Block: All
