# Supplementary material for: Perceptions of Adult Obesity Education: A Pilot Study
Source: J Med Educ Curric Dev. 2024 Oct 1;11:23821205241269371. doi: 10.1177/23821205241269371 (PMC11450567; doi:10.1177/23821205241269371)
Supplement: sj-docx-2-mde-10.1177_23821205241269371 - Supplemental material for Perceptions of Adult Obesity Education: A Pilot Study [file sj-docx-2-mde-10.1177_23821205241269371.docx]

# PROTOCOL TITLE:

**UCD PRIMARY CARE ADULT OBESITY**

**TRAINING & TREATMENT SERVICES ASSESSMENT**

**PRINCIPAL INVESTIGATOR**: Seleda Williams, MD, MPH, VCF

**RESEARCH ASSISTANT:** Cara Sandholdt, PhD

# KEY INFORMANT INTERVIEW TOOL OBESITY CURRICULUM & TRAINING

Introduction

Hello “Administrator”. Is it ok to call you by your first name? My name is Dr.

Seleda Williams and I am here with my assistant Cara Sandholdt. We are both Scholars with the UCD Interprofessional Teaching Scholars Program (ITSP) and this research is part of my ITSP research project. My research assistant will be helping me with taking notes of our discussions. I am a UCD Volunteer Clinical Faculty member with the Departments of Internal Medicine and Public Health. Thank-you for meeting with us today for an interview about UCD adult obesity curriculum and training for the Betty Irene Moore School of Nursing which I will refer to as (SON) family nurse practitioner and physician assistant (FNP/PA) students, who I will refer to as FNP or PA students.

Our interview today is a conversation about what you think about the obesity curriculum and clinical training for SON FNP/PA students, and it will consist of 12 core questions. In addition to the 12 core questions, I might also ask some additional questions to clarify what we are discussing. We will try to keep the total time to one hour.

I would like to give you a brief overview of this research study. The reason for

this study is to find out what curricula and clinical training on adult obesity is currently being offered to UCD SON FNP/PA students as well as UCD FM/IM residents and how UCD obesity training and primary care treatment services could be improved. This will be done by 1.) administering electronically a Qualtrics survey and 2.) by conducting face to face or video key informant interviews with four to five UCD primary care residency and nursing school administrators. Your participation is related to part 2. Your opinions and recommendations are very important to the quality of this study and will help improve training and services for adult obesity within the UCD outpatient primary setting.

Let me now tell you about our target populations. For purposes of this

research study, UCD “primary care” refers to UCD Internal Medicine (IM) Faculty and Residents; UCD Family & Community Medicine (FM) Faculty and Residents; and the UCD Betty Irene School of Nursing (SON) Family Nurse Practitioners (FNP)/Physician Assistants (PA) Faculty and students.

Thank-you for completing our consent forms. Just to confirm with you these

interviews are voluntary, and your name and titles will be removed from the all transcripts and reports. You will be given and ID number instead and referred to as an Administrator. Interviews will also be recorded and transcribed. You may ask not to be

recorded during the interview. We may also ask you to review our summary of the transcripts for accuracy.

So, let’s get started with our interview questions. This is just a reminder that we have 12 core questions to discuss and any other items that come up.

Questions for Key Informants

1. When I say obesity curriculum for UCD FNP OR PA students what comes to mind to you?
2. Tell me about what curriculum on adult obesity is currently being offered to UCD PA OR SON FNP students?
3. What clinical training on adult obesity, such as clinical rotations, is currently being offered to UCD FNP/PA students [select appropriate department].
4. Please tell me about the American Nurses Credentialing Center or ANCC and/or the American Academy of Nurse Practitioner or AANP OR [or National Commission on Certification of Physician Assistants (NCCPA}] exam requirements on the topic of obesity.
5. What types of additional curricula would you recommend for SON PA OR nursing students? What do you see as some of the barriers? Facilitators?
6. What are your thoughts about the education of UCD PA OR FNP students on lifestyle medicine topics such as nutrition, as it relates to obesity; physical activity, as it relates to obesity; and behavioral health? Do you see any other challenges or positives? What about OBESITY DRUGS?
7. What types of additional clinical training would you recommend for nursing/PA students on weight management for adult obese patients? What do you see as some of the barriers? Are there any positive aspects in clinical training at UCD on adult obesity?
8. Do you have any additional thoughts on how obesity curricula and teaching be improved at UCD SON FNP/PA students [select Department]?
9. What are your thoughts about a course on obesity for UCD nursing/PA students, such as a “Obesity 101”? Elective course versus compulsory course? Any barriers?
10. Is there anything else I should know or that I didn’t ask about adult obesity training?
11. We’ve talked about curriculum and training on adult obesity, but I’m curious to know what your thoughts are on current obesity treatment services at the UCD healthcare center?
12. Is there anyone else you think I should speak with?

I want to thank-you very much for taking the time today to assist us with our research. Please feel free to contact me if you have any further questions or concerns. We will provide you a copy of our interview summary when available.

Please feel free to contact Cara and me if you have any further questions: Dr. Seleda Williams, MD, MPH. [drswilliams@ucdavis.edu](mailto:drswilliams@ucdavis.edu)

[Probing Questions]: If further clarification is needed, such as questions as: What did you mean by this? Could you explain your comments further? Let me clarify what I meant by obesity curriculum.
