# Supplementary material for: Perceptions of Adult Obesity Education: A Pilot Study
Source: J Med Educ Curric Dev. 2024 Oct 1;11:23821205241269371. doi: 10.1177/23821205241269371 (PMC11450567; doi:10.1177/23821205241269371)
Supplement: sj-docx-3-mde-10.1177_23821205241269371 - Supplemental material for Perceptions of Adult Obesity Education: A Pilot Study [file sj-docx-3-mde-10.1177_23821205241269371.docx]

UCD PRIMARY CARE ADULT OBESITY

UCD ADMINISTRATORS: KEY INFORMANT INTERVIEWS

| **CATEGORIES** | **ADMINISTRATOR A.1**  **UCD School of Nursing, Physician Assistant Program** |
| --- | --- |
| **Curriculum** | Definition: What does obesity curriculum mean to you? Obesity is a problem in our country, and we need to do a better job teaching about nutrition in primary care. Students need to advocate for patients.  Providers need to be more knowledgeable. Economic issues are important and can impact the ability to treat obese patients. Need societal changes.  Offerings   - Some nutrition during cardiovascular - Not sure about whether there is a dedicated specific curriculum on obesity. If it is there it is limited. - Not sure about coverage of obesity medications. May be covered in the Pharmacology class. |
| **Clinical Experience** | Current Offerings:   - On primary care rotations PA students are seeing patients with obesity issues. It depends on the rotation as to how much exposure the students get. - Mental Health rotation is a requirement but not specific to obesity. - Some students do bariatric surgery rotations. This is usually where PA students would get most content on obesity regarding the morbidly obese meeting criteria for surgery. |
| **Barriers** | - Not having a point person on faculty for obesity teaching. - Finding time in the curriculum. There is already so much to teach. - Need to go through NPPA sub-committee and then Education Policy Curriculum Development (EPCD) Committee and graduate committees. Office of Graduate studies committee reviews and approves which takes more time. - Need more dedicated content on obesity. - Need to know more about what works to treat obesity. - This should be expanded in the motivational interviewing and education of patients’ aspect of the curriculum |
| **Opportunities** | - In the process of revising curriculum for second year. Could add content on management of obesity. Add competencies and resources. - First year Medicine 251 series could add more content on obesity. 4^th^ Quarter (251D, summer) special populations might also offer opportunities for expanding obesity content. |

|  | - Just extended one of their courses (251D, summer) from 5-6 to10 weeks, which could offer some time to teach obesity. - In future they may have separate courses on pediatrics and women’s health and that might allow for instruction on pediatric obesity. - Course learning outcomes are kept broad which may allow moving around curriculum content. - Could add content in behavioral health. Provides leeway in curriculum. - Developing a Doctor of Nursing program (DNP) which may provide opportunities for AP curriculum revamp and adding   more content on obesity. |
| --- | --- |
| **Credentialing** | - Accrediting body requires more specifics on competencies. Physician Assistant National Certifying Exam (PANCE) is the certification exam, which covers a variety of topics. Patient education and counseling are part of the requirements. There may be just a few questions on obesity. - Objective Structured Clinical Exams (OSCE), always have some cases with high BMI but more focused on co-morbidities of obesity. - UCD also has a written summative exam for PA students. Education and counseling are covered on this exam. - UCD was planning on using the new PAEA comprehensive exam (available) in August 2020 but has been delayed due to coronavirus pandemic. |
| **Additional** | - Lifestyle Medicine: well set up to discuss diet and exercise. These topics are discussed in cardiology. Would have to check on whether mental health topics cover behavioral management of weight loss. Would be nice to do so. Students can rotate with MD, PA, NP, LCSW plus or minus MD. Rotating with an RD would be useful. - Not sure about how much is covered on obesity medications. - “Obesity 101” type course: PAs do not have didactive electives, but they do have selective rotations in second year. Not sure that a full course would work. Incorporating content   into other courses would be better. |
| **Treatment Services** | - Does not work in the hospital so not sure what is currently being offered. |
| **Recommendations** | - Having a more dedicated course on obesity. - Content on obesity could be added into other courses such as medicine (251 series). - Add more training on obesity in the second year. - Look at OSCEs as a way to add more content about obesity. Look at a case of an obese patient with pre-diabetes. Add obesity management to OSCE competencies. - Appoint a point person on faculty as an expert on obesity who can help develop the curriculum. - Combine the topic with other cases such as hypertension, diabetes, etc. Family issues. |

|  | - Not an entire course on obesity but expand opportunities within current curriculum and clinical rotations. - Consider modules on nutrition and lifestyle. |
| --- | --- |

| **CATEGORIES** | **ADMINISTRATOR A.2: Betty Irene Moore School of Nursing**  **Family Nurse Practitioner (FNP) Program** |
| --- | --- |
| **Curriculum** | Definition: What does obesity curriculum mean to you? A combination of risk factors, genetics, common assumptions, energy, nutritional content, lifestyle modification, psychosocial content, environment, treatment. Challenging the assumptions about obesity. Professor from Internal Medicine Nutrition Clinic comes and lectures students about obesity.  Offerings: limited, not sure of all content being offered.   - SON is developing Doctor of Nursing Program (DNP) curriculum and this may be an area where they could weave in obesity training into the course focused on illness. - Teaching modules could be helpful. |
| **Clinical Experience** | Current Offerings:   - First year FNP is primarily didactic and second year is primarily clinical. - Some content on metabolic syndrome, diabetes, hypertension, hyperlipidemia with a look at lifestyle modification. - Some offerings with training on endocrine disorders such hypothyroidism and depression. - Some clinical cases on obesity. - Other than Family Practice rotations “that would be it”. - Not fully comprehensive. |
| **Barriers** | - Based on multiple facets of obesity not sure that we know how to teach patients how to maintain a healthy weight. - Not having enough time in the curriculum. - Getting new course content through the UC system. - Course content on obesity curriculum is not clearly defined. |
| **Opportunities** | - Look at literature related to obesity as a disease. - Work on changing the perspective of the provider that if you are overweight it is because you are eating too much. - Ideal would be to have a weight management clinical rotation. - Will be revising NP/PA curriculum in the next couple of months, this will be an opportunity to look at obesity curriculum and rotation opportunities. - Look at and expand content on energy metabolism. - Looking at going live with a UCD Doctorate of Nurse Practitioner hybrid program in 2022. Could provide opportunity to develop didactics on obesity, online modules and clinical rotation or clinical case studies. - Ideally all FNPs should have course or rotation on obesity. |

| **Credentialing** | - American Nurses Credentialing Center or ANCC and/or the American Academy of Nurse Practitioner or AANP OR [or National Commission on Certification of Physician Assistants (NCCPA) exam requirements on the topic of obesity: - Most likely minimal content on these exams, however this is an opportunity to review current competency requirements on obesity. - Focus is on obesity’s contribution to disease. |
| --- | --- |
| **Additional** | - Need more on lifestyle medicine, maintaining healthy weight, stress reduction., and trauma informed care (impact of adverse childhood events (ACDS). However, lifestyle topics are covered in guidelines for diabetes and endocrine disorders. - Not sure about the depth of obesity content being offered. Currently in the process of curriculum mapping. - Need to also look at training related to pediatric obesity. - Need more training on pediatric obesity as well. |
| **Treatment Services** | - Not sure about treatment services. |
| **Recommendations** | - Teach more on the etiology of obesity, nutrition, energy metabolism, genetic components. - Need to look more at what is covered in FNP certification requirements on obesity. This can help faculty by providing knowledge. - Need to look at what is being offered in the Advanced Pharmacology course on obesity drugs. Need to coordinate with pharmacology. - Examine course objectives and revise. - Offer clinical rotation on weight management. - Certain courses such as Preparation for Clinical Practice and Foundations of Behavioral Health could add various topics related to obesity. - An “obesity 101” course could be considered. |

| **CATEGORIES** | **ADMINISTRATOR A.3: UCD Internal Medicine (IM) Residency Program** |
| --- | --- |
| **Curriculum** | Definition: What does obesity curriculum mean to you? “I think of having a curriculum that is more robust than what we have.” Regarding primary care obesity curriculum, it would include initial assessment, available treatments, nutritionist, dietitians, low calorie diets, medication management and surgical interventions. Good at diagnosing but not good at utilizing resources.  Offerings   - No formalized curriculum on obesity. - Not sure if there are any seminars being offered. - Do offer John Hopkins module on obesity is available to IM residents. |

| **Clinical Experience** | Current Offerings:   - Not exactly sure of what is being offered for didactics. No specific IM clinical rotations. - Some residents rotate through the UCD IM Nutrition Clinic. - IM residents have their own continuity panel of 50-100 patients and usually have half day sessions. - Residents gain experience managing patients with obesity in primary care clinics. They have a basic understanding of metabolic syndrome. About 20% of IM residents go into Primary Care. - Most IM residents go into subspecialties such as cardiology, pulmonary critical care, GI, hem oncology and a few specialize in endocrinology, allergies, and rheumatology. Also a few residents are hospitalists. - Very important for those residents who go into IM primary care. - Residents get exposed to the co-morbidities of obesity. - They know how to refer patients, but they need more fundamental knowledge and clinical experience on the   treatment of obesity. |
| --- | --- |
| **Barriers** | - Limited time in class or clinical rotations to teach about obesity because IM must cover all internal medicine subjects. - Limited opportunities for formal classes versus other teaching formats such as modules. - Struggle with residents completing learning modules. - Limitation of faculty who are comfortable talking about obesity. - Do not have a robust clinical program on obesity. - There is a lack of referral programs. - Need more curriculum on pharmacologic management. Medications are not usually covered by health insurance or difficulty to get authorized. - There have been financial limitations in starting a very low- calorie diet program. This is an area where primary care IM providers could play a role - Even if a VLCD program is offered there are not than many IM patients that can afford the program. |
| **Opportunities** | - IM has 1-2 junior faculty members who are interested in starting an obesity management clinic but need experience in financial modeling. - IM trains well on obesity related co-morbidities, such as diabetes, obstructive sleep apnea and metabolic syndrome. This can be an opportunity to look at how obesity treatment can be incorporated in training. - There is considerable information in the community now about wellness and mindfulness that residents and providers are aware of. |
| **Credentialing** | - USMLE Step 3: not sure if there are specific competencies for obesity. There may be a question or two on IM Board   examination but not considered a critical competency. |

| **Additional** | - About 100 residents need to be considered. Lifestyle Medicine: - They probably struggle more with nutritional assessment. - They also get basics on exercise assessment and guidelines. - Residents do get behavioral health seminar series and go to med-psych clinic rotation. Deal with topics such as depression but not necessarily behavioral health management of obesity. - An “Obesity 101” type course is hard to do in residency training program. Might be easier to do in the nurse practitioner and physician assistant programs. |
| --- | --- |
| **Treatment Services** | - Does not appear to be an organized UCD obesity management program. However, there is a bariatric surgery program. - Aware that UCLA had a dedicated physician where residents could train on obesity. - Refers to UCD LifeSteps program with some of patients being successful. - UCD registered dietitians are available. - Need better longitudinal care. - Difficult to find treatment who are rejected from bariatric surgery because their BMI is too high. Not sure where to send these patients. |
| **Recommendations** | - Utilize nutritionist to assist with building didactic. - Consider short online learning opportunities. Having well defined modules is important. - Could explore providing some seminars. - Offer additional clinical rotations such as the ones offered in the IM Nutrition/Weight Management Clinic. Also offering a two-week block elective rotation 4-5 half day s a week. - Offer opportunity to rotate through the bariatric surgery clinic. - There should be tailored training where Primary Care IM residents would get more in-depth training and Specialty IM   residents would get more tailored training. |

| **CATEGORIES** | **ADMINISTRATOR A.4: UCD Family & Community Medicine (FM) Residency Program** |
| --- | --- |
| **Curriculum** | Definition: What does obesity curriculum mean to you? Offerings   1. FM residency is based on topic- based curriculum, in formal once weekly in person lectures, interactive workshops or other types of teaching. 2. There may not be a formal based topic on obesity, however, it may be covered under other FM topics. 3. Typically do an 18-month curriculum. |

|  | There are two main types of training:   - Didactic, classroom, asynchronous, online and in person Tuesday workshops, interactive sessions. However not sure if specific training on obesity is offered to FM residents. - Direct clinical care working with patients.   Topics covered include preventive health, obesity and adult co- morbidities, pediatric obesity. |
| --- | --- |
| **Clinical Experience** | Current Offerings:   - No formal clinical training on obesity. - Psychologist is available for training on motivational interviewing. - Past weight management clinic needs dedicated faculty and financial viability. Typically had high no show rates. |
| **Barriers** | - Trying to make everything fit in with the many other demands to address various required topics. - Previously had a faculty with a weight management clinic but had difficulty getting residents to attend this clinic. Need accessible, trained faculty. - Finances: justifying that it is cost effective. Not enough revenues. - Need something that covers all bases rather than an ongoing module. - No obesity department or center where residents could do some training, other than Nutrition Clinic. |
| **Opportunities** | - Fully in support of offering additional training on obesity. Would be beneficial. |
| **Credentialing** | - USMLE Step 3 credentialing exams National test is not specialty driven. - Suspect there are obesity questions, but not sure of specifics. Probably not a full section on about obesity on USMLE Step 3 exams. - FM Board Exams: Most likely a few questions but broader in nature. |
| **Additional** | - Lifestyle medicine: Have a behavioral psychologist, however not a huge area of content need. - In future, topics on lifestyle medicine, such as nutrition, physical activity, behavioral health, etc. might be covered under a single topic. Embed all in a single case module or clinical vignette. - “Lifestyle medicine is what we do.” |
| **Treatment Services** | - Bariatric surgery clinic is available at UCD. - Registered dietitians area available. - Could we do more for an obesity clinic. Sure. - Past FM weight management clinic problem was that there were not enough revenues. - May not be a big demand right now because of coronavirus pandemic. |

| **Recommendations** | - Recommend contacting USMLE to get further information about USMLE exam questions on obesity. - Online module about obesity would be well received. - One- hour interactive cases or clinical vignette are good. - Adding a topic on obesity in 18- month curriculum is reasonable. - Having a half- day course or rotation for about 2-4 weeks, 2-4 times a week that a resident could attend. - Offering an obesity “101” type course as an elective is a possibility. FM residents have 12 weeks of elective over a 3- year training period. May be more useful at medical school level. - Clinical focus would be more useful to FM residents. What to do when a patient walks into a room. |
| --- | --- |
