# Supplementary material for: Perceptions of Adult Obesity Education: A Pilot Study
Source: J Med Educ Curric Dev. 2024 Oct 1;11:23821205241269371. doi: 10.1177/23821205241269371 (PMC11450567; doi:10.1177/23821205241269371)
Supplement: sj-docx-4-mde-10.1177_23821205241269371 - Supplemental material for Perceptions of Adult Obesity Education: A Pilot Study [file sj-docx-4-mde-10.1177_23821205241269371.docx]

**Supplement 4: Equator COREQ CHECKLIST**

Consolidated criteria for reporting qualitative studies (COREQ): 32-item checklist

| **No** | **Item** | **Guide questions/description** |
| --- | --- | --- |
| **Domain 1: Research team and reflexivity** |  |  |
| Personal Characteristics |  |  |
| 1. | Interviewer/facilitator | Which author/s conducted the interview or focus group?   - ***Due to anonymous reviews author’s names were not included in the manuscript*** |
| 2. | Credentials | What were the researcher's credentials? *E.g. PhD, MD*   - *Listed in the Title Page & Submission site* |
| 3. | Occupation | What was their occupation at the time of the study?   - *Listed in the Title page & Submission site* |
| 4. | Gender | Was the researcher male or female?  N/A |
| 5. | Experience and training | What experience or training did the researcher have?   - *Degrees and educational affiliations listed on Title page.* |
| Relationship with participants |  |  |
| 6. | Relationship established | Was a relationship established prior to study commencement?   - *A relationship was established with the program directors and administrative staff* |
| 7. | Participant knowledge of the interviewer | What did the participants know about the researcher? e*.g. personal goals, reasons for doing the research*   - *Key Informant Interviewers were introduced to the researchers. Survey participants did not have knowledge about the researchers.* |
| 8. | Interviewer characteristics | What characteristics were reported about the interviewer/facilitator? e.g. *Bias, assumptions, reasons and interests in the research topic*   - *Participants were informed by email and phone about the intent of the research and reasons why the researchers requested the interviews. Informed consent was required.* |
| **Domain 2: study design** |  |  |
| Theoretical framework |  |  |
| 9. | Methodological orientation and Theory | What methodological orientation was stated to underpin the study? *e.g. grounded theory, discourse analysis, ethnography, phenomenology, content analysis*   - *Logic Model methodology was used to develop research study.* |
| Participant selection |  |  |
| 10. | Sampling | How were participants selected? *e.g. purposive, convenience, consecutive, snowball*   - *Convenience sample* |
| 11. | Method of approach | How were participants approached? e*.g. face-to-face, telephone, mail, email*   - *Email invitation and telephone calls . Invitations were sent inviting participation in a webinar* |
| 12. | Sample size | How many participants were in the study?   - *Four in the key informant interviews: Interviewees were the Directors of their delegates of the primary care programs including Family Medicine, Internal Medicine, Family Nurse Practitioner and Physician Assistant training programs.* |
| 13. | Non-participation | How many people refused to participate or dropped out? Reasons?   - *None dropped out from the key informant interview invitations.* |
| Setting |  |  |
| 14. | Setting of data collection | Where was the data collected? e*.g. home, clinic, workplace*   - *Via WebEx video interview* |
| 15. | Presence of non-participants | Was anyone else present besides the participants and researchers?   - *No* |
| 16. | Description of sample | What are the important characteristics of the sample? *e.g. demographic data, date*   - *Key informant interviews were administrators of the relevant research groups* |
| Data collection |  |  |
| 17. | Interview guide | Were questions, prompts, guides provided by the authors? Was it pilot tested?   - *A set of questions were developed for the interviewees. The interviews were part of a larger pilot study, which also included a survey.* |
| 18. | Repeat interviews | Were repeat interviews carried out? If yes, how many?   - *No repeat interviews were carried out. However, all key informant interviewees were invited to review the theme tables developed by the researchers.* |
| 19. | Audio/visual recording | Did the research use audio or visual recording to collect the data?   - *Yes, video interviews and recordings. Informed consent was required.* |
| 20. | Field notes | Were field notes made during and/or after the interview or focus group?   - *Yes. Handwritten as well as WebEx video transcripts were available* |
| 21. | Duration | What was the duration of the interviews or focus group?   - *Length of interviews were about one hour* |
| 22. | Data saturation | Was data saturation discussed?   - *No* |
| 23. | Transcripts returned | Were transcripts returned to participants for comment and/or correction?   - *Theme tables were provided to all interviewees and access to video interviews were available upon request.* |
| **Domain 3: analysis and findings**z |  |  |
| Data analysis |  |  |
| 24. | Number of data coders | How many data coders coded the data?   - *Two data coders. These the PI and Co-PI* |
| 25. | Description of the coding tree | Did authors provide a description of the coding tree?   - *No, a coding tree was not developed. Thematic analysis was conducted based on the interview questions and interviewee responses and a Table created summarizing the themes.* |
| 26. | Derivation of themes | Were themes identified in advance or derived from the data?   - *Yes, some themes were anticipated, but some themes were developed based upon the participant responses.* |
| 27. | Software | What software, if applicable, was used to manage the data?   - *No software was used for the interview portion of this research study.* |
| 28. | Participant checking | Did participants provide feedback on the findings?   - *Yes, all agreed on thematic analysis* |
| Reporting |  |  |
| 29. | Quotations presented | Were participant quotations presented to illustrate the themes / findings? Was each quotation identified? e*.g. participant number*   - *Yes. Key informant interview select quotations were provided. To help provide anonymity a participant number was not provided.* |
| 30. | Data and findings consistent | Was there consistency between the data presented and the findings?   - *Yes* |
| 31. | Clarity of major themes | Were major themes clearly presented in the findings?   - *Yes, major themes were presented in the findings and the key theme tables provided as a supplement.* |
| 32. | Clarity of minor themes | Is there a description of diverse cases or discussion of minor themes?   - *Some minor themes were discussed in the paper, but no description of diverse cases was provided.* |
