# Supplementary material for: Perceptions of Adult Obesity Education: A Pilot Study
Source: J Med Educ Curric Dev. 2024 Oct 1;11:23821205241269371. doi: 10.1177/23821205241269371 (PMC11450567; doi:10.1177/23821205241269371)
Supplement: sj-docx-5-mde-10.1177_23821205241269371 - Supplemental material for Perceptions of Adult Obesity Education: A Pilot Study [file sj-docx-5-mde-10.1177_23821205241269371.docx]

**Consensus Reporting Items for Studies in Primary Care CRISP Checklist**

The CRISP Checklist^1^ can help researchers meet readers’ needs by including content that the primary care community feels is important for the validity, quality, and usefulness of primary care research reports.

The Checklist summarizes recommendations for authors to consider in crafting their report. Nothing is intended to limit the effective or creative reporting of research. Authors and editors make final decisions.

Primary care research involves a wide variety of methods, study designs, topics, and settings. The Checklist covers this broad spectrum and therefore, not all items apply to all studies. Some items may not apply to your study. Some may ask for information that is not available. Check such items off and use the rest of the Checklist in whatever way helps you improve *your* report of *your* research.

How to use the CRISP Checklist:

- Each item is listed. Please respond to each item, even if it is not included in your report.
- Check if the item is included in your report: yes, no, or not applicable to your study.
- If the item applies to the study design but is not included in the report, please provide a brief explanation in the “Notes” section.
- The “Suggested Section” indicates where the item usually appears in a research report following the IMRaD format (Introduction, Methods, Results, and Discussion). These are suggestions only. If the item is in a different section of your report, you might add that in “Notes.”
- You can note the location of the item in your report by line, page, or section in “Notes.”

For more information plus an explanation and examples of each item, please see the supplemental guidance in the Appendix to the CRISP Statement.^2^

You may choose to submit the completed Checklist along with your manuscript to help editors and reviewers see how you have included the suggested items in your research report. Authors should also consider using other reporting guidelines that are appropriate for their study and report. (See Table 2.^1^) Some CRISP items may overlap with other guidelines.

For more information, see **CRISP:** <https://crisp-pc.org/>

**References:**

1. Improving the Reporting of Primary Care Research: Consensus Reporting Items for Studies in Primary Care—the CRISP Statement. William R. Phillips, Elizabeth Sturgiss, Paul Glasziou, Tim C. olde Hartman, Aaron M. Orkin, Pallavi Prathivadi, Joanne Reeve, Grant M. Russell, and Chris van Weel. *Annals of Family Medicine* November 2023, 21 (6) 549-555; DOI: <https://doi.org/10.1370/afm.3029>
2. Supplemental Appendix. Consensus Reporting Items for Studies in Primary Care (CRISP) Explanation and Examples Guide. <https://www.annfammed.org/content/annalsfm/suppl/2023/10/02/afm.3029.DC1/Phillips-Supp-App-Table-2023.pdf>

| **Reporting Item - 1** | **Included?** | | | **Section*** | | **Notes** |  |
| --- | --- | --- | --- | --- | --- | --- | --- |
|  | **Y** | **N** | **N/A** |  |  | | |
| **1. Include “primary care” and/or discipline-specific terms in the title, abstract, and/or key words.** | **X** |  |  | I | Discussed in Abstract and manuscript. Included as a key word. Survey was titled: “Adult Obesity Primary Care Survey” | | |

| **Reporting Item - 2** | **Included?** | | | **Section*** | **Notes** |
| --- | --- | --- | --- | --- | --- |
|  | **Y** | **N** | **N/A** |  |  |
| **2. Describe the study rationale and importance for primary care.** | | | | | |
| **2a.** Explain the rationale for the research question and how it relates to primary care. | **X** |  |  | I  D | Discussed in the Introduction & Discussion sections |
| **2b.** Describe the importance or relevance of the topic under study in the primary care setting. | **X** |  |  | I  D | Discussed in the Introduction & Discussion sections |
| **2c.** Identify any theory, model, or framework used, and explain why it is appropriate to the research question in primary care. | **X** |  |  | I | This research was a pilot study. A logic model was developed early in the research process. Hypotheses and specific aims/objectives were developed. |

| **Reporting Item - 3** | **Included?** | | | **Section*** | **Notes** |
| --- | --- | --- | --- | --- | --- |
|  | **Y** | **N** | **N/A** |  |  |
| **3. Describe the research team’s primary care experience and collaboration.** | | | | | |
| **3a.** Describe the research team’s expertise and experience in primary care practice and/or research. | **X** |  |  | T  M | Identified in the Title Page and Methods sections. |
| **3b.** Describe whether and how primary care patients, practicing clinicians, community members, or other stakeholders were involved in the research process. | **X** |  |  | M | Discussed in the Methods section |

| **Reporting Item - 4** | **Included?** | | | **Section*** | **Notes** |
| --- | --- | --- | --- | --- | --- |
|  | **Y** | **N** | **N/A** |  |  |
| **4. Describe the study participants and populations in the context of primary care.** | | | | | |
| **4a.** Use person-focused language to refer to the research populations and participants, or use terms based on patient preferences | **X** |  |  | M  R | Person focused language used with respect to the study population and obesity related care. |
| **4b.** If reporting personal characteristics of participants, report the source of the data, the rationale for using it, and the rationale for any classifications used. |  |  | **X** |  |  |
| **4c.** Describe the participants and populations in sufficient detail to allow comparison to other primary care patient populations. | **X** |  |  | M  R | Academic participants and level of training identified for all research participants. This was not a patient study. |
| **4d.** Specify whether participants have pre-existing therapeutic relationships with the clinical team or are new patients. |  |  | **X** | M/R | This study was not a clinical intervention. It examined primary care administrator, faculty and trainee perceptions about their training on adult obesity. |

| **Reporting Item - 5** | **Included?** | | | **Section*** | **Notes** |
| --- | --- | --- | --- | --- | --- |
|  | **Y** | **N** | **N/A** |  |  |
| **5. Describe the conditions under study in the context of primary care.** | | | | | |
| **5a.** Describe whether the condition under study is acute or chronic. |  |  | **X** | M/R | Not applicable. This study included a survey and key informant interviews |
| **5b.** Report how multimorbidity is considered and how it might affect interpretation of the study findings/ results. |  |  | **X** | M | Multimorbidity was evaluated in the context of multi-disciplinary training. |

| **Reporting Item - 6** | **Included?** | | | **Section*** | **Notes** |
| --- | --- | --- | --- | --- | --- |
|  | **Y** | **N** | **N/A** |  |  |
| **6. Describe the clinical encounter under study in the context of primary care.** | | | | | |
| **6a.** Specify whether the study focus is an isolated clinical encounter or a longitudinal course of care. If it is an isolated clinical encounter, specify whether it is the first visit or a follow-up visit for the condition under study |  |  | **X** | M | This study was a survey of primary care administrators, faculty, FM/IM residents and FNP/PA students and key informant interviews of relevant primary care FM/IM and FNP/PA directors or their delegates. It examined their perceptions about their confidence and training on adult obesity. |

| **Reporting Item - 7** | **Included?** | | | **Section*** | **Notes** |
| --- | --- | --- | --- | --- | --- |
|  | **Y** | **N** | **N/A** |  |  |
| **7. Describe the patient care team.** | | | | | |
| **7a.** If care is delivered by teams, describe the team members and their roles. | **X** |  |  | M  R | Interprofessional teams were described. Professional roles were identified. Included primary care, clinical nutrition, physical activity, behavioural health, bariatric surgery |
| **7b.** For each clinician category, report profession, specialty, and qualifications. | **X** |  |  | M  R | Clinical professions were identified. Family Medicine, Internal Medicine, Family Nurse Practitioners and Physician Assistants. Level of training identified. |

| **Reporting Item - 8** | **Included?** | | | **Section*** | **Notes** |
| --- | --- | --- | --- | --- | --- |
|  | **Y** | **N** | **N/A** |  |  |
| **8. Describe the study interventions in the context of primary care.** | | | | | |
| **8a.** Describe interventions and their implementation in sufficient detail to enable the reader to assess applicability in their own setting. |  |  | **X** | M | This was not a clinical intervention study. The interventions in this study were key informant interviews and survey distribution. |
| **8b.** Describe any clustering or grouping of patients, participants, clinicians, teams, or practices, and how it was addressed in the analysis. | **X** |  |  | M/R | The study design and description of the key informant interviews and survey participants were clearly outlined in the study. The Methods section describe how survey participants were clustered and described in the Results. |
| **8c.** Describe the health care system in sufficient detail to allow comparisons to other systems. | **X** |  |  | I/D | The Introduction outlined the type of institution and participants evaluated. The Future Directions section under the Discussion looked at how the study could be expanded to other institutions. |

| **Reporting Item - 9** | **Included?** | | | **Section*** | **Notes** |
| --- | --- | --- | --- | --- | --- |
|  | **Y** | **N** | **N/A** |  |  |
| **9. Describe study measures used and their relevance to primary care.** | | | | | |
| **9a.** Report whether study measurement tools have been validated in primary care populations or settings. | **X** |  |  | M | The Limitations section reported that this study was a pilot and did not validate the survey or interview questions. |
| **9b.** Describe how the measurement tools used are meaningful to primary care patients and their care. | **X** |  |  | M  D | The survey and key informant interviews identified areas where primary care practitioners need more training in the assessment and treatment of adult obesity. Specific multi-disciplinary areas were identified. |
| **9c.** Report findings/results to be clinically interpretable by primary care clinicians and patients. | **X** |  |  | R  D | This study was not a clinical intervention. However, the survey and key informant interview results are highly relevant to clinical practice for primary care trainees, clinicians, and relevant educators. |

| **Reporting Item - 10** | **Included?** | | | **Section*** | **Notes** |
| --- | --- | --- | --- | --- | --- |
|  | **Y** | **N** | **N/A** |  |  |
| **10. Discuss the meaning of study findings/results in the context of primary care.** | | | | | |
| **10a.** Discuss implications of the study findings/results for research, patient care, education, and policy with specific focus on primary care. | **X** |  |  | D | This pilot study addresses education and policy issues as it relates to training on adult obesity in primary care training programs. |
| **10b.** Discuss the implications of study recommendations on demands and priorities in primary care practice. | **X** |  |  | D | This study discussed the implications and future directions for primary care training on adult obesity. |
| **10c.** Comment on any research processes that might influence the applicability of the study findings/results in diverse primary care settings. | **X** |  |  | D | This was discussed in the Discussion section including Limitations and Future Directions. |

***Section:** I = Introduction, M = Method, R = Results, D = Discussion
